# Supplementary material for: Effects of Eggshell Thickness, Calcium Content, and Number of Pores in Erosion Craters on Hatching Rate of Chinese Alligator Eggs
Source: Animals (Basel). 2023 Apr 19;13(8):1405. doi: 10.3390/ani13081405 (PMC10135048; doi:10.3390/ani13081405)
Supplement: Supplementary file 1 [file animals-13-01405-s001.zip › animals-2258030-supplementary.pdf]

Supplementary Table S1. Normal Test Table for Number of erosion crater pores and Thickness at Cracking End, Middle End, and Complete End of Eggshell

	Number of erosion crater pores			Thickness		
	CE	ME	IE	CE	ME	IE
statistic	0.195	0.144	0.238	0.079	0.088	0.062
df	80	80	80	80	80	80
Sig.	0.000	0.003	0.000	0.200	0.200	0.200

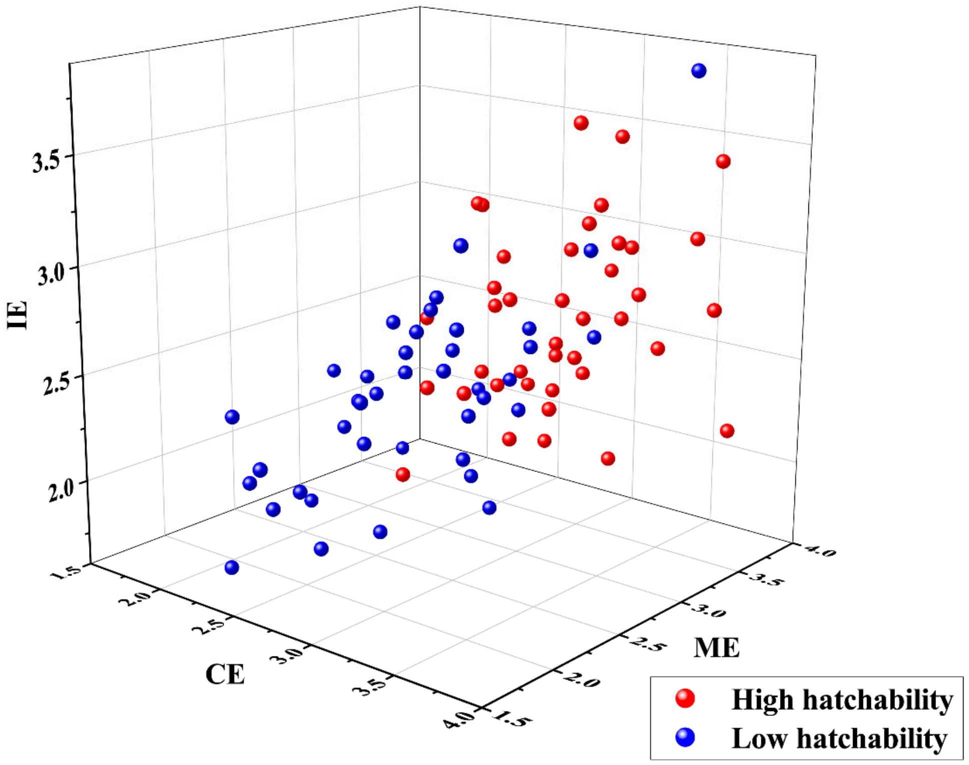

Supplementary Fig. S1. 3D map of average eggshell thickness

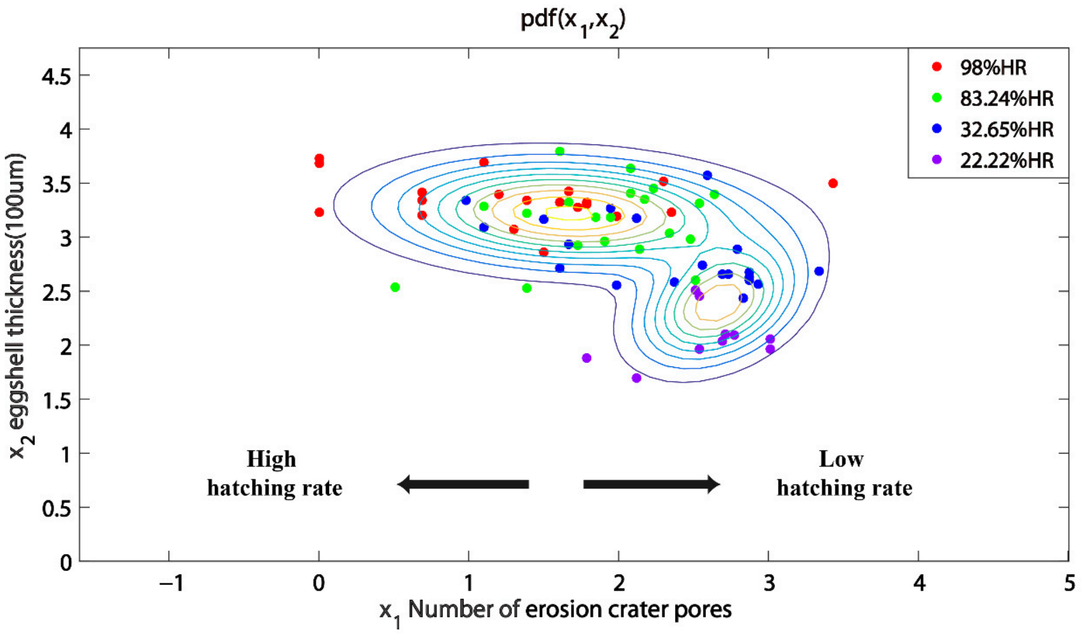

Supplementary Fig. S2. ME Gaussian Mixture Clustering Model

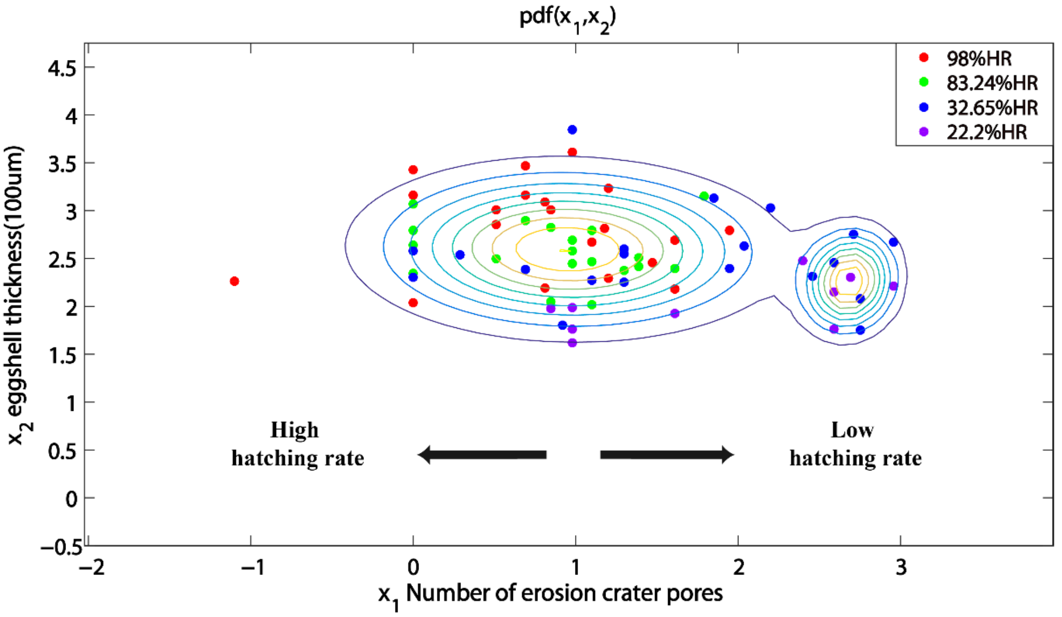

Supplementary Fig. S3. IE Gaussian Mixture Clustering Model

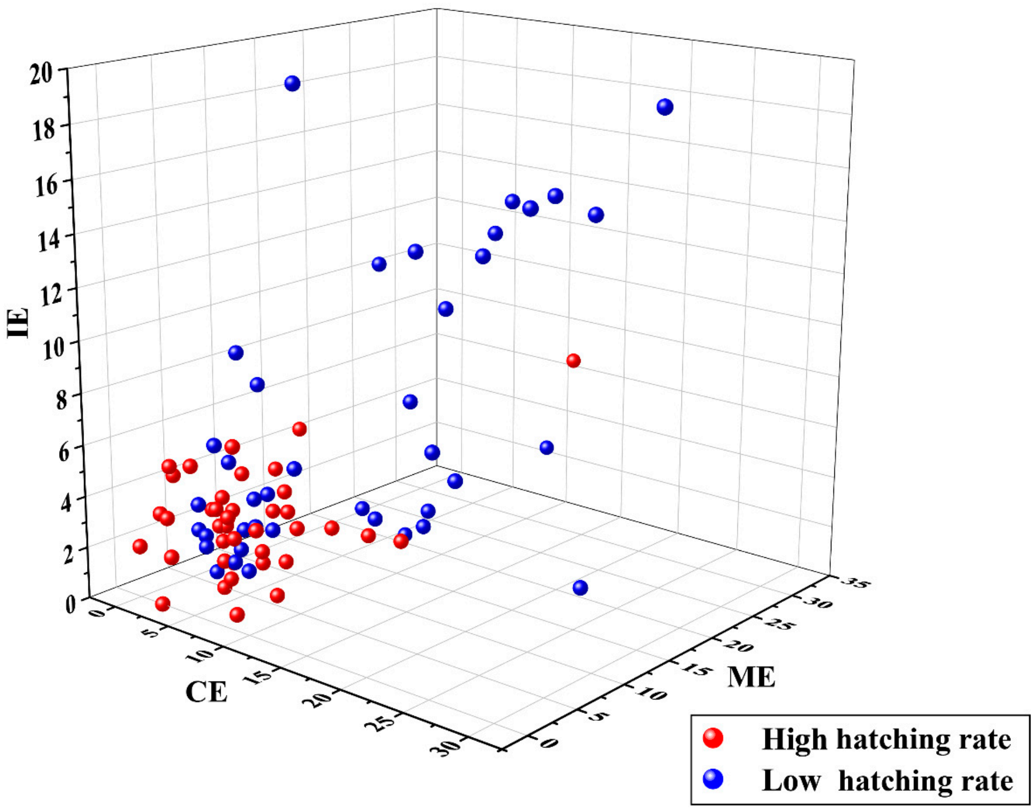

Supplementary Fig. S4. 3D map of average the number of erosion crater pores

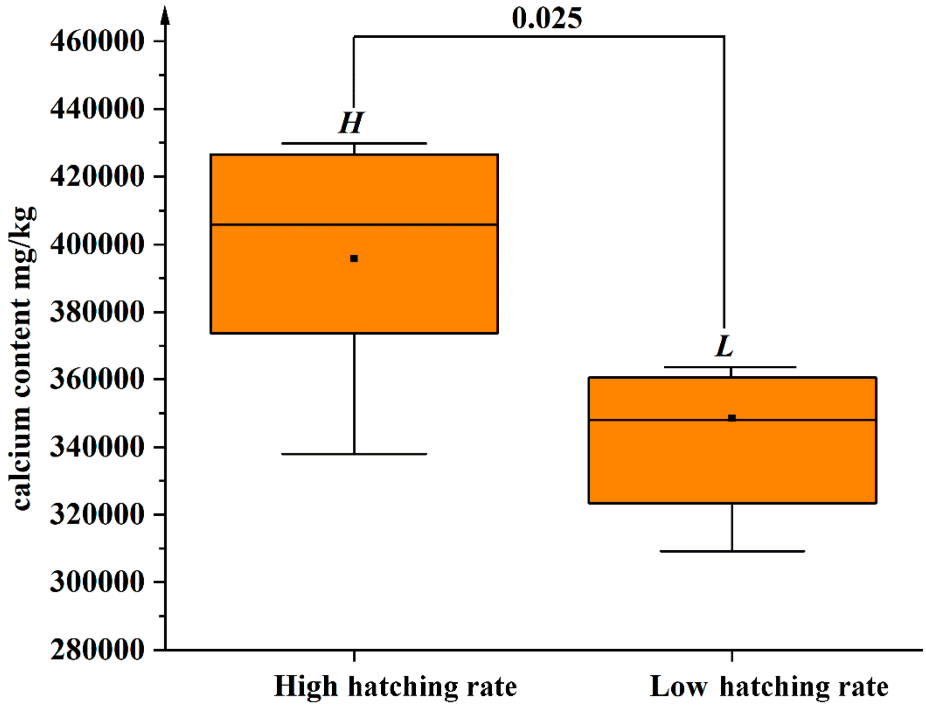

Supplementary Fig. S5. Calcium content in eggshells with different hatching rates
